# Supplementary material for: Development of a hepatic cryoinjury model to study liver regeneration
Source: bioRxiv. 2024 Jun 17:2023.07.24.550437. Preprint. [Version 2] doi: 10.1101/2023.07.24.550437 (PMC11212901; doi:10.1101/2023.07.24.550437)
Supplement: Supplement 1 [file NIHPP2023.07.24.550437v2-supplement-1.pdf]

bioRxiv preprint doi: https://doi.org/10.1101/2023.07.24.550437; this version posted June 17, 2024. The copyright holder for this preprint (which was not certified by peer review) is the author/funder, who has granted bioRxiv a license to display the preprint in perpetuity. It is made available under aCC-BY 4.0 International license.

Supplementary Figures

bioRxiv preprint doi: https://doi.org/10.1101/2023.07.24.550437; this version posted June 17, 2024. The copyright holder for this preprint (which was not certified by peer review) is the author/funder, who has granted bioRxiv a license to display the preprint in perpetuity. It is made available under aCC-BY 4.0 International license.

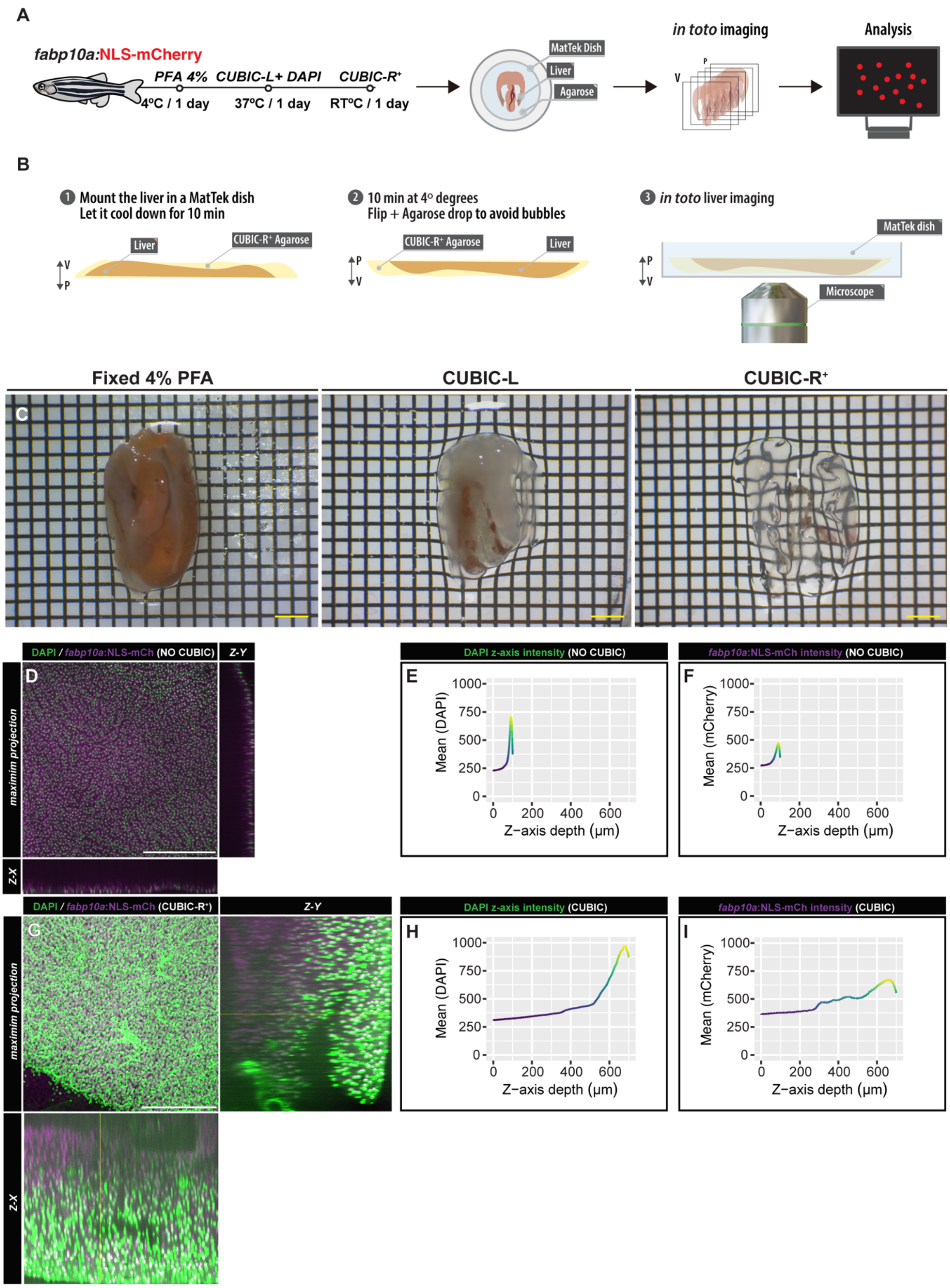

Supplementary Figure 1. CUBIC-clearing liver method. A) Schematic representation of the liver CUBIC-clearing procedure for imaging the liver. B) Mounting strategy for imaging cleared livers. Livers are embedded in CUBIC-agarose within a MatTek dish, positioning the IA facing-up. The CUBIC-agarose disc containing embedded livers is cooled down for 10 minutes at 4 degrees Celsius. Once the CUBIC-agarose disc solidifies, it is carefully flipped using forceps, and a drop of CUBIC-agarose is added to prevent bubble formation. Cleared livers are prepared for imaging from this point onwards.

bioRxiv preprint doi: https://doi.org/10.1101/2023.07.24.550437; this version posted June 17, 2024. The copyright holder for this preprint (which was not certified by peer review) is the author/funder, who has granted bioRxiv a license to display the preprint in perpetuity. It is made available under aCC-BY 4.0 International license.

C) An example of the liver-clearing process during the CUBIC-clearing procedure. D and G) z-stack representative examples, with Z-X and Z-Y projections, of non-cleared (D) and CUBIC-cleared (G) *in toto* liver confocal acquisitions. E-F) Mean intensity of DAPI (E) and mCherry (F) along the z-axis in non-cleared *in toto* liver confocal acquisition. H-I) Mean intensity of DAPI (H) and mCherry (I) along the z-axis in CUBIC-cleared *in toto* liver confocal acquisition. Scale bars: 2 mm (yellow), 500 µm (white).

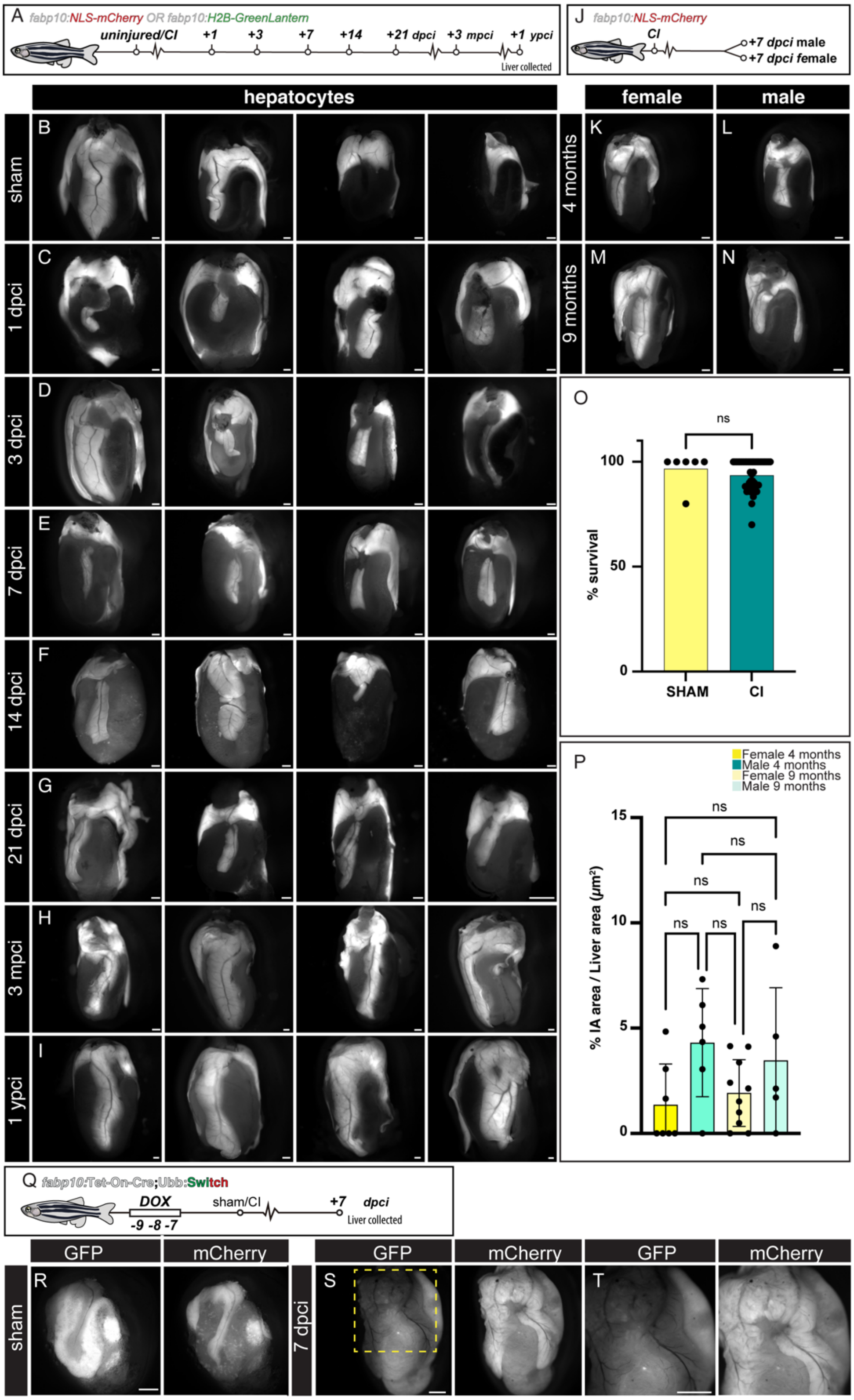

bioRxiv preprint doi: https://doi.org/10.1101/2023.07.24.550437; this version posted June 17, 2024. The copyright holder for this preprint (which was not certified by peer review) is the author/funder, who has granted bioRxiv a license to display the preprint in perpetuity. It is made available under aCC-BY 4.0 International license.

Supplementary Figure 2. Temporal dynamics of liver regeneration in zebrafish following cryoinjury. A) Schematic representation of the timing of sample collection following cryoinjury. B-I) Epifluorescence acquisitions of adult zebrafish Tg(*fabp10a: GreenLantern-H2B*) or Tg(*fabp10a:NLS-mCherry*) livers at different stages of regeneration upon cryoinjury. J) Schematic representation of the timing of sample collection following cryoinjury between female and male adult zebrafish. K-N) Epifluorescence acquisitions of Tg(*fabp10a:NLS-mCherry*) livers in adult zebrafish differentiate between females (K and M) and males (L and N), as well as between those aged 4 (K-L) months and 9 months (M-N), at 7 dpci. O) Zebrafish survival upon cryoinjury (n= 444; survival= 92.97 %; *p*-value: 0.5843 unpaired Student's *t*-test). P) Quantification of the IA area (n= 6, 4, 8, 5), bars indicate mean and SD, *p*-values: one-way ANOVA followed by Tukey's multiple comparisons test). Q) Schematic representation of the timing of recombination and collection following cryoinjury. R-T) Epifluorescence acquisitions of adult zebrafish Tg(*fabp10a: Tet-ON-Cre; Ubb:Switch*) at sham and 7 dpci. CI: cryoinjury; Dox: Doxycline; dpci: day post-cryoinjury; mpci: months post-cryoinjury; ypci: years post-cryoinjury. Scale bars: 500 µm (B-I and K-N) and 1000 µm (R-T).

bioRxiv preprint doi: https://doi.org/10.1101/2023.07.24.550437; this version posted June 17, 2024. The copyright holder for this preprint (which was not certified by peer review) is the author/funder, who has granted bioRxiv a license to display the preprint in perpetuity. It is made available under aCC-BY 4.0 International license.

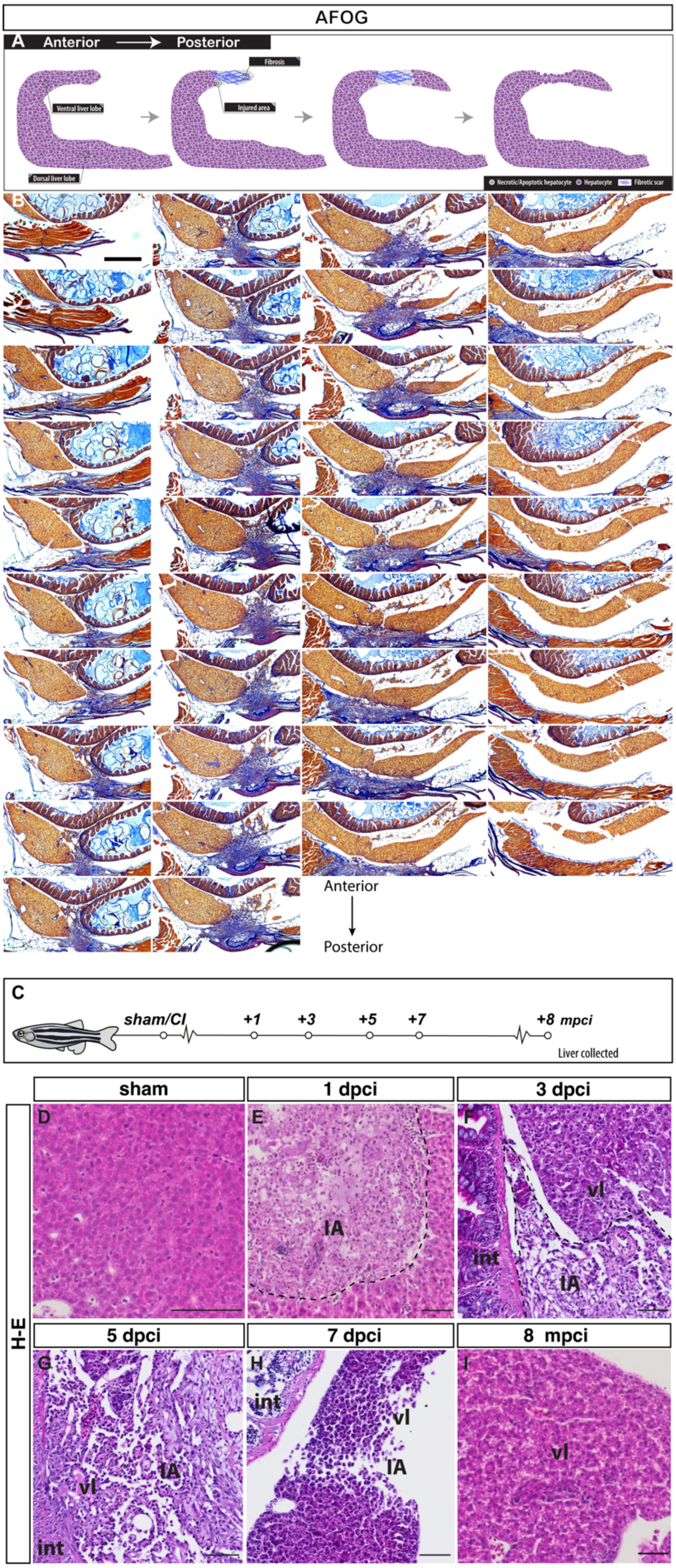

bioRxiv preprint doi: https://doi.org/10.1101/2023.07.24.550437; this version posted June 17, 2024. The copyright holder for this preprint (which was not certified by peer review) is the author/funder, who has granted bioRxiv a license to display the preprint in perpetuity. It is made available under aCC-BY 4.0 International license.

Supplementary Figure 3. AFOG staining and histological appearance of regenerating livers following cryoinjury. A) Schematic representation of the histology section plane across 5 dpci adult zebrafish liver. B) AFOG staining of consecutive sections of 5 dpci adult zebrafish liver. Anterior is towards the left, dorsal is towards the top. C) Representation of the timing of sample collection following cryoinjury for histological examination. D-I) H&E staining of adult zebrafish liver, sham (D), 1 day post-cryoinjury (dpci; E), 3 dpci (F), 5 dpci (G), 7 dpci (H), and 8 months post-cryoinjury (mpci; I). Blue: collagen; Red: cell debris and fibrin; IA: injured area; int: intestine; vl: ventral lobe. Scale bars: 500 µm (B) and 50 µm (D-H).

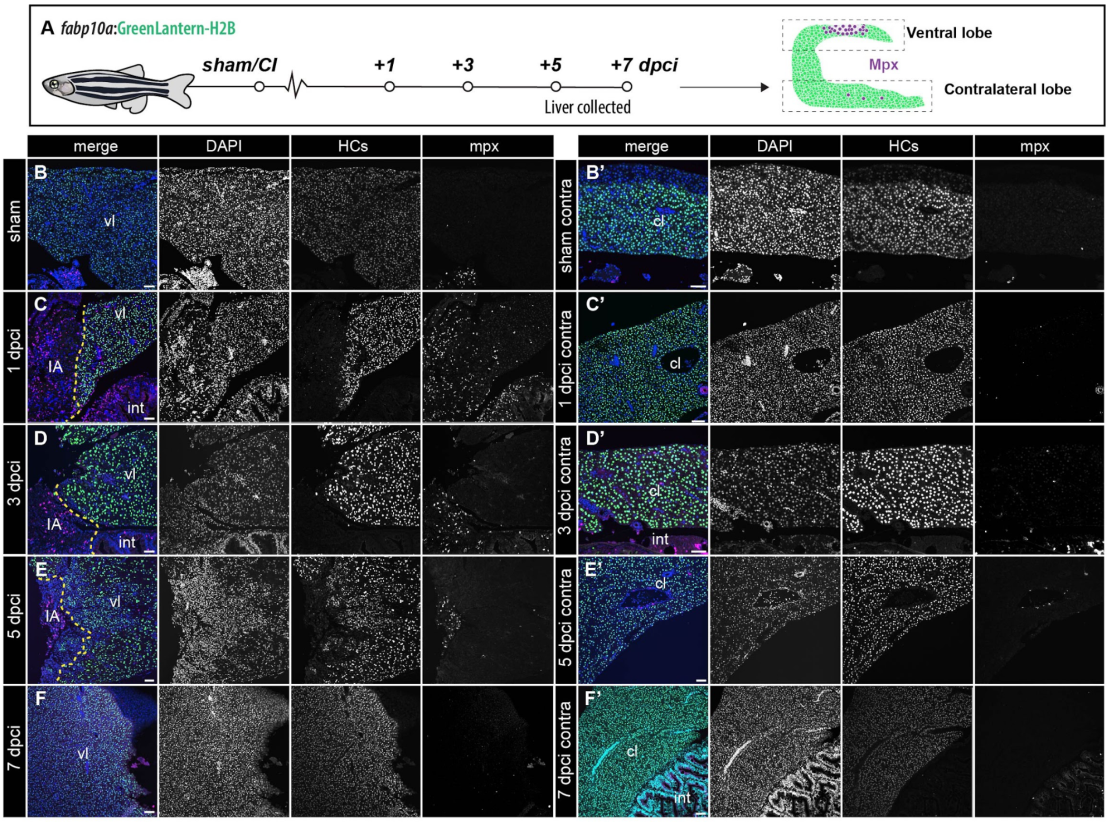

Supplementary Figure 4. Mpx⁺ Neutrophils dynamics during liver regeneration upon cryoinjury. A) Schematic representation of the experiment workflow. B-F) Sections of livers from *Tg(fabp10a*:GreenLantern-H2B*)* animals at the indicated stages in ventral lobes compared to contralateral ones, immunostained to detect hepatocyte nuclei (GFP) and leukocytes (Mpx). cl: contralateral lobe; Dashed yellow line: border zone; IA: injured area. Scale bars: 50 µm.

bioRxiv preprint doi: https://doi.org/10.1101/2023.07.24.550437; this version posted June 17, 2024. The copyright holder for this preprint (which was not certified by peer review) is the author/funder, who has granted bioRxiv a license to display the preprint in perpetuity. It is made available under aCC-BY 4.0 International license.

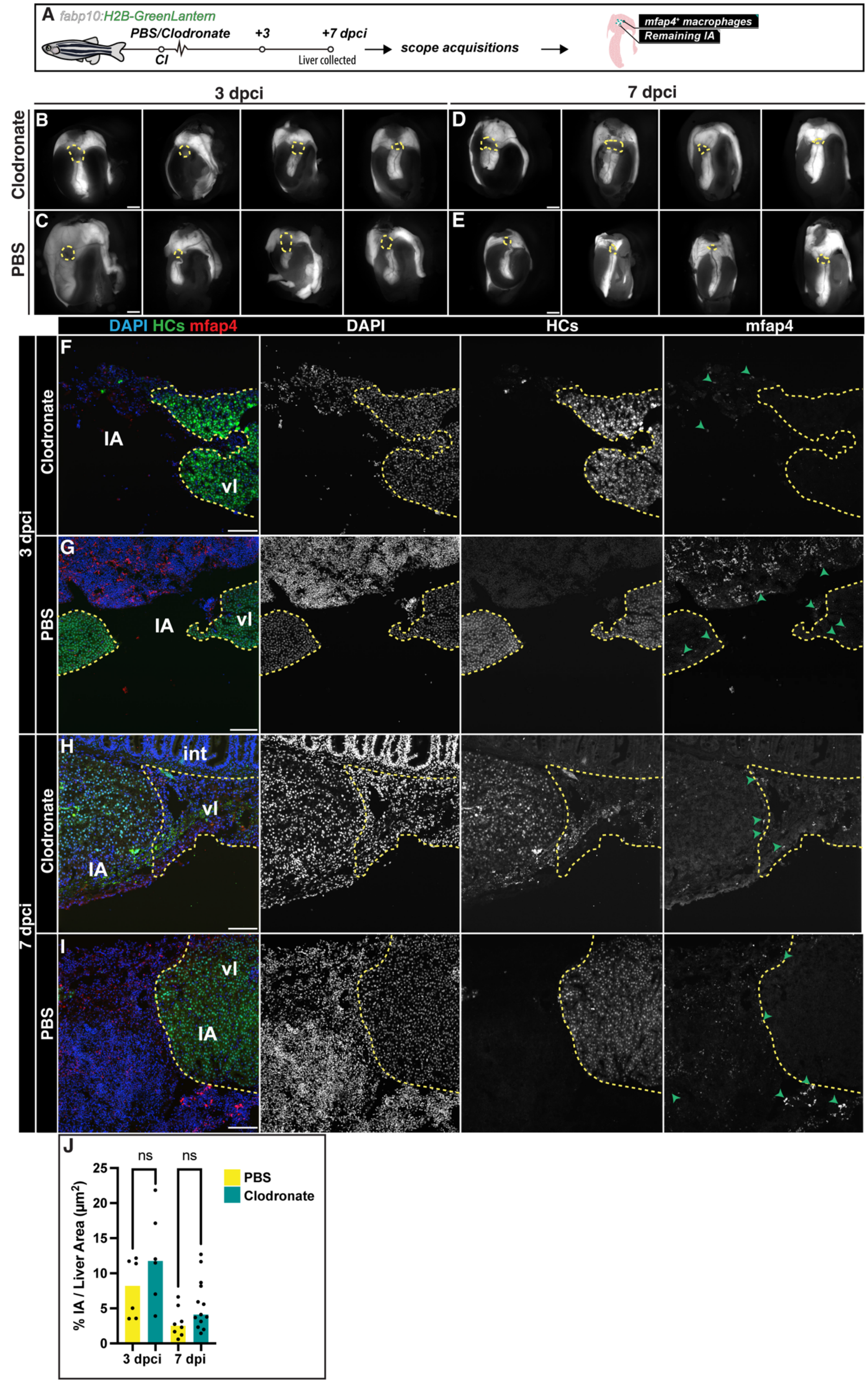

bioRxiv preprint doi: https://doi.org/10.1101/2023.07.24.550437; this version posted June 17, 2024. The copyright holder for this preprint (which was not certified by peer review) is the author/funder, who has granted bioRxiv a license to display the preprint in perpetuity. It is made available under aCC-BY 4.0 International license.

Supplementary Figure 5. Clodronate effects on macrophages dynamics during liver regeneration upon cryoinjury. A) Schematic representation of the timing of clodronate injection and sample collection following cryoinjury. B-E) Epifluorescence acquisitions of adult zebrafish of Tg(*fabp10a:*H2B-GreenLantern) livers at 3 dpci with clodronate and controls with PBS IP injections (B-C), and 7 dpci with clodronate and PBS IP injections (D-E). F-I) Adult liver sections from cryoinjured animals at 3 dpci (F-G) and 7 dpci (H-I), immunostained to detect HCs nuclei (GreenLantern) and macrophages (mfap4) in clodronate injected samples (F and H), and PBS injected controls (G and I). J) Quantification of the IA area (n= 6, 6, 8, 13), bars indicate median, *p*-values: one-way ANOVA followed by Tukey's multiple comparisons test. Dashed line: Border zone of the injured area; IA: injured area; int: intestine; vl: ventral lobe. Scale bars: 500 µm (B-E), and 100 µm (F-I).

bioRxiv preprint doi: https://doi.org/10.1101/2023.07.24.550437; this version posted June 17, 2024. The copyright holder for this preprint (which was not certified by peer review) is the author/funder, who has granted bioRxiv a license to display the preprint in perpetuity. It is made available under aCC-BY 4.0 International license.

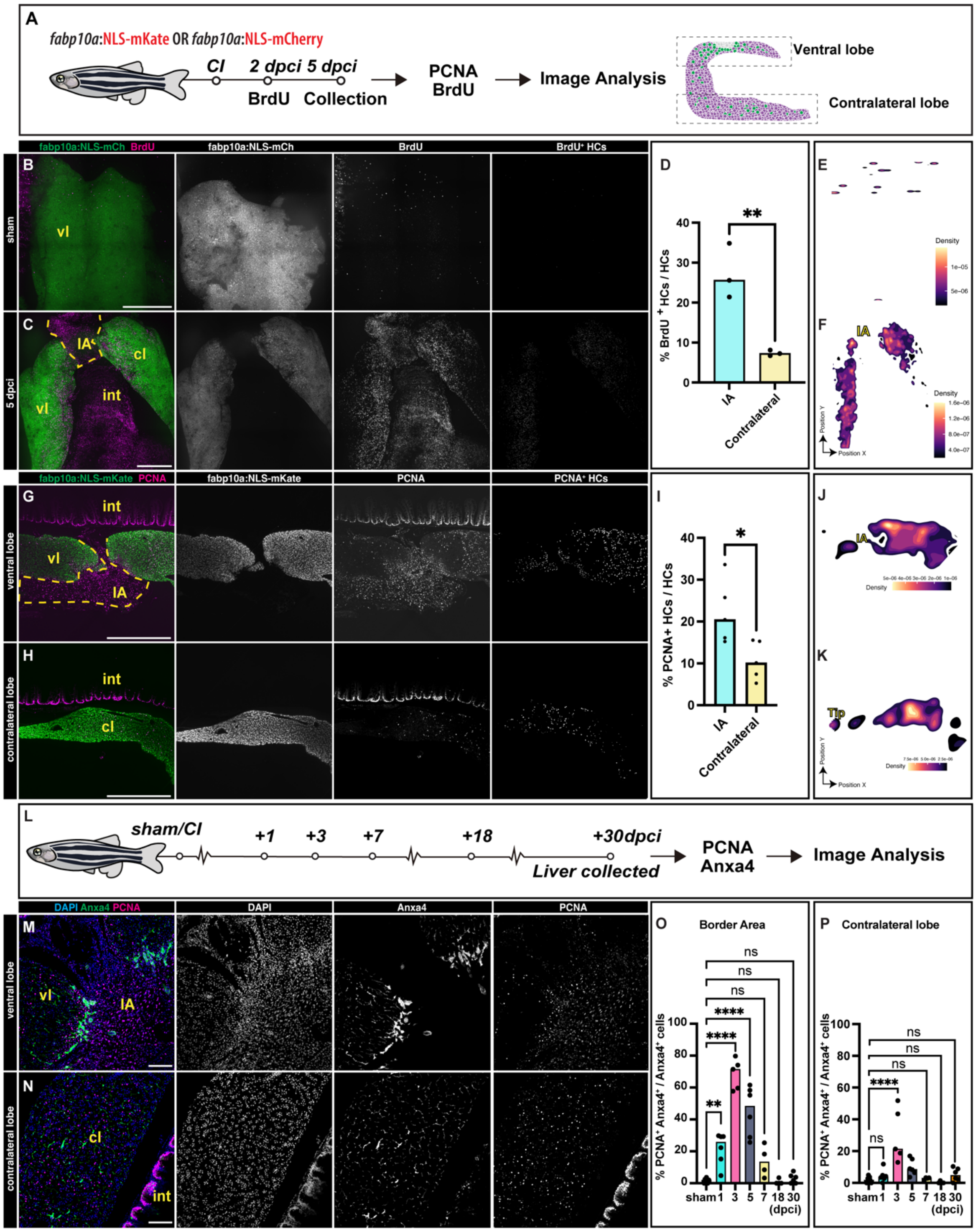

Supplementary Figure 6. Cryoinjury induces local and distal compensatory hyperplasia. A) Schematic representation of the timing of sample collection following cryoinjury for BrdU and PCNA analysis. B) *in toto* of BrdU-pulse labelling sham adult zebrafish confocal acquisition. C) *in toto* of BrdU-pulse labelling 5 dpci adult zebrafish confocal acquisition. D) Quantification of BrdU$^+$ mCherry$^+$ HCs vs total number of mCherry$^+$ HCs in the ventral lobe and contralateral one, bars indicate median, *p*-value: unpaired Student's *t*-test. E-F) *in toto* confocal acquisition segmentation of BrdU$^+$/mCherry$^+$ HCs nuclei and density analysis using a Kernel Density Estimation (KDE) algorithm presented as a corresponding heatmap and quantification of the proliferating HCs density in sham animals (E) and 5 dpci (F). G) *in toto* of PCNA stained 5 dpci adult zebrafish confocal acquisition at the ventral lobe. H) *in toto* of PCNA stained 5 dpci adult zebrafish confocal acquisition at the contralateral lobe. I)

bioRxiv preprint doi: https://doi.org/10.1101/2023.07.24.550437; this version posted June 17, 2024. The copyright holder for this preprint (which was not certified by peer review) is the author/funder, who has granted bioRxiv a license to display the preprint in perpetuity. It is made available under aCC-BY 4.0 International license.

Quantification of PCNA⁺ mKate⁺ HCs vs total number of mKate⁺ HCs in the ventral lobe and contralateral one, bars indicate median, *p*-value: unpaired Student's *t*-test. J-K) *in toto* confocal acquisition segmentation of PCNA⁺/mKate⁺ HCs nuclei and density analysis using a Kernel Density Estimation (KDE) algorithm presented as a corresponding heatmap and quantification of the proliferating HCs density in the ventral lobe (J) and contralateral lobe (K). L) A simplified schematic illustrating the collection of livers following cryoinjury for PCNA⁺ and Anxa4⁺ biliary epithelial cells (BECs). M-N) Adult liver sections from cryoinjured animals at 5 dpci, immunostained to detect proliferation (PCNA) and BECs (Anxa4). O-P) Quantification of PCNA⁺ Anxa4⁺ BECs vs total number of BECs in the injured border area (O) vs contralateral lobe (P) from the indicated cohorts, (n = 6, 5, 5, 6, 4, 4 and 6; error bars representing SD; *p*-values: one-way ANOVA followed by Tukey's multiple comparisons test). Dashed line: Border zone of the injured area; dpci: days post-cryoinjury; IA: injured area; int: intestine; vl: ventral lobe. Scale bars: 500 µm.

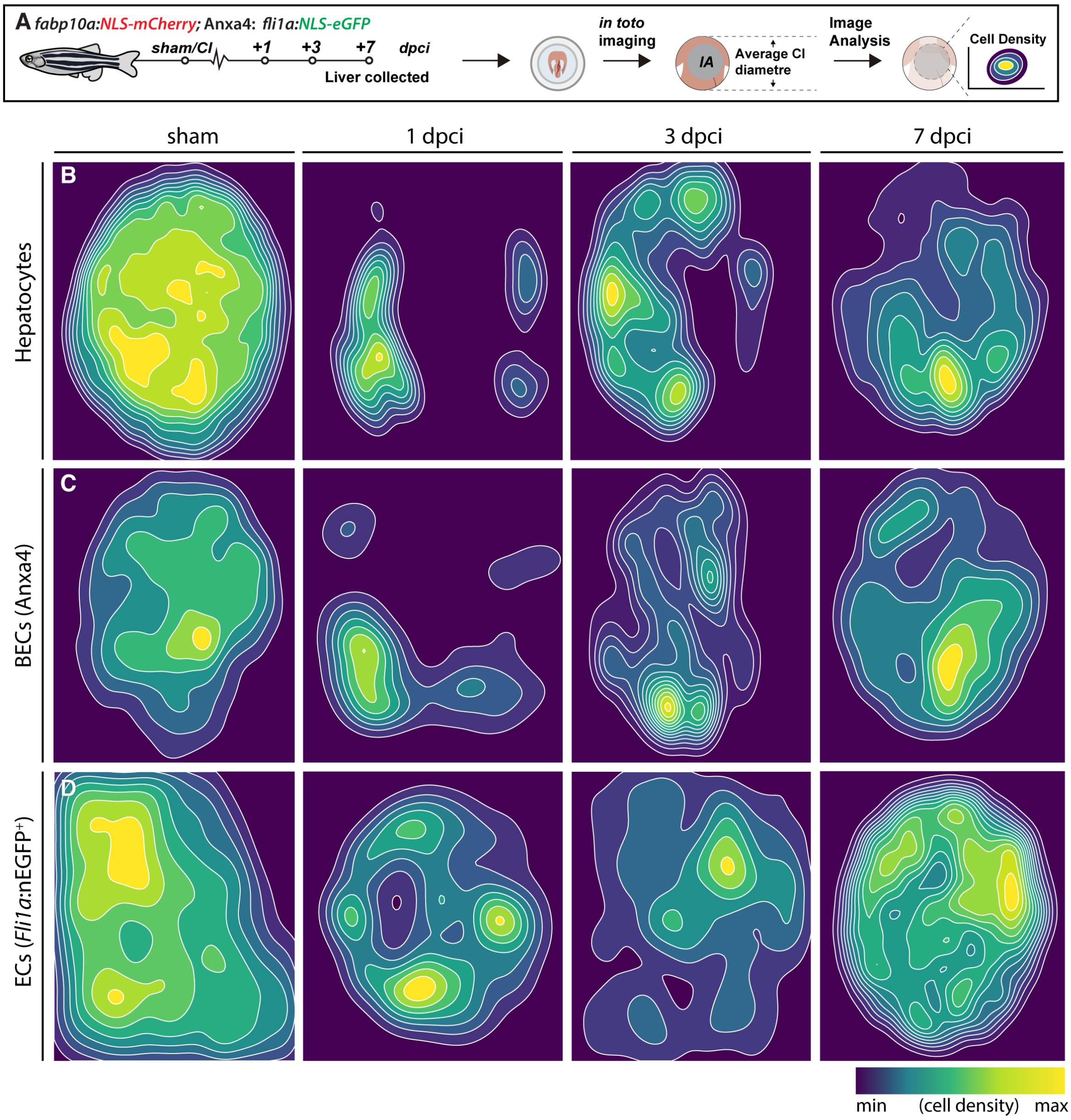

Supplementary Figure 7. Regeneration of the injured area. A) Schematic workflow from data acquisition to image analysis. B-D) Region of interest (ROI) represents the integration of four independent datasets within the average injured area size of adult livers. (B) Tg(*fabp10a*:NLS-mCherry; n: 4), (C) immunostained against Anxa4 (n: 4), (D) Tg(*fli1a*:NLS-eGFP; n: 4) zebrafish. The probabilistic

bioRxiv preprint doi: https://doi.org/10.1101/2023.07.24.550437; this version posted June 17, 2024. The copyright holder for this preprint (which was not certified by peer review) is the author/funder, who has granted bioRxiv a license to display the preprint in perpetuity. It is made available under aCC-BY 4.0 International license.

location of HCs (B), BECs (C), and ECs (D) is represented as a density plot over the first seven days of liver repair upon cryoinjury.

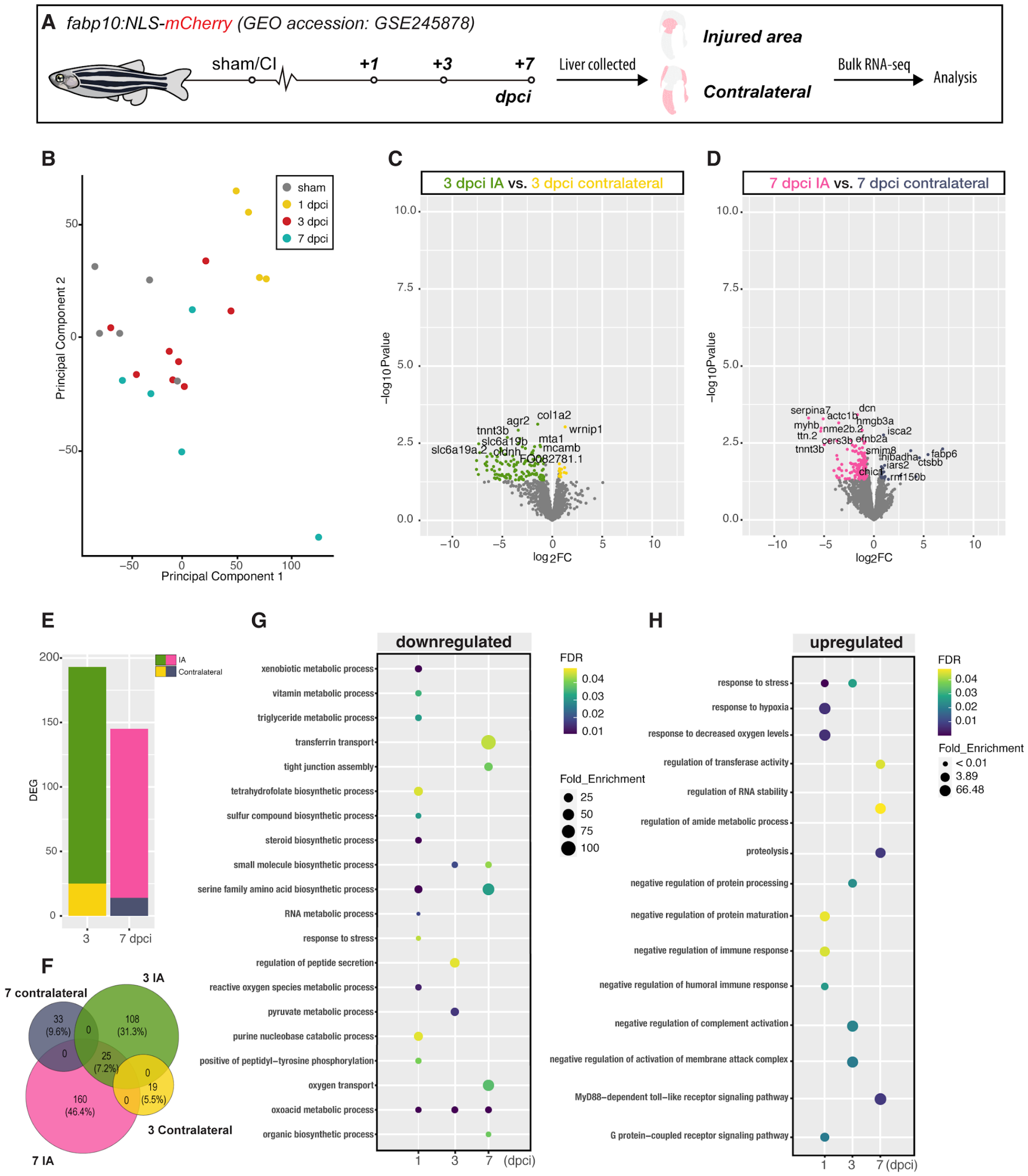

Supplementary Figure 8. Comprehensive Gene Ontology (GO) Analysis of Regenerating Liver Tissue. A) Graphical illustration of the collection process (IA or Contralateral lobes) for liver repair stages following cryoinjury and sequencing. B) Principal Component Analysis (PCA) of sham-operated and injured ventral lobes at different stages including sham, 1, 3, and 7 dpci (Technical replicates = 4 per timepoint; livers = 16 per timepoint). C-D) Volcano plot representing Bulk RNA-seq data from the IA compared to Bulk RNA-seq data from contralateral liver lobes at 3 (C) and 7 (D) dpci. DEGs (FC ≥1.5 (Yellow or Navy Blue) or ≤-1.5 (Green or Pink); *P*≤0.05) with top DEG annotated. E) Bar plot representing the number of upregulated and downregulated DEG in IA and contralateral lobes at 3 and 7 dpci. F) Venn diagram representing DEG in IA and contralateral lobes at 3 and 7 dpci. G-H) GO-

bioRxiv preprint doi: https://doi.org/10.1101/2023.07.24.550437; this version posted June 17, 2024. The copyright holder for this preprint (which was not certified by peer review) is the author/funder, who has granted bioRxiv a license to display the preprint in perpetuity. It is made available under aCC-BY 4.0 International license.

enrichment analysis of IA Bulk RNA-seq data showing both downregulated (G) and upregulated (H) terms during liver regeneration following cryoinjury in the IA of the ventral lobe.

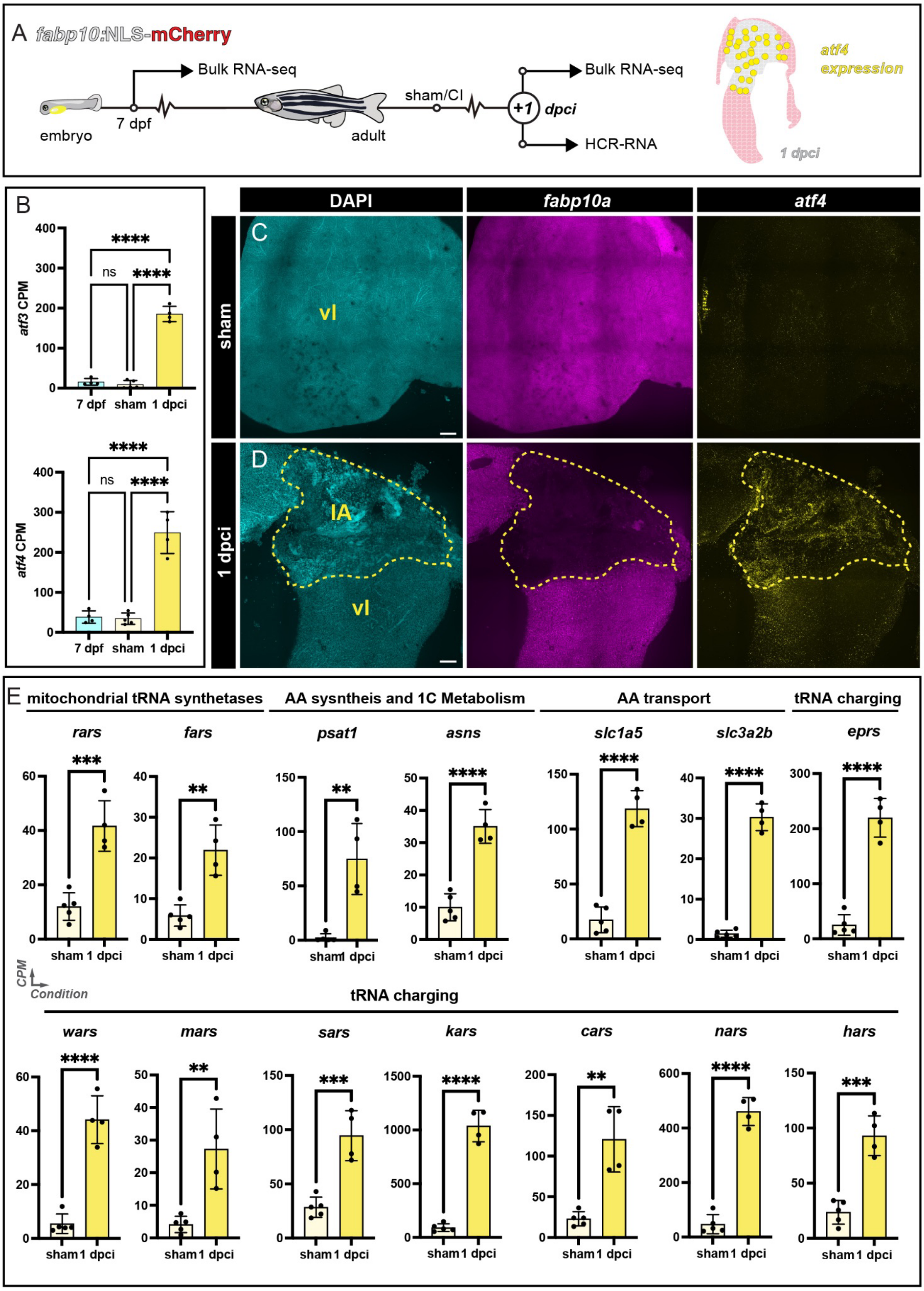

Supplementary Figure 9. Upregulation of the Integrated stress response (ISR) upon cryoinjury in the zebrafish. A) Graphical illustration of the collection process for sequencing. B) atf4 and atf3 CPM of Bulk RNA-seq livers during developmental (7 dpf), sham and 1 dpci samples. C-D) fapb10a

bioRxiv preprint doi: https://doi.org/10.1101/2023.07.24.550437; this version posted June 17, 2024. The copyright holder for this preprint (which was not certified by peer review) is the author/funder, who has granted bioRxiv a license to display the preprint in perpetuity. It is made available under aCC-BY 4.0 International license.

and atf4 HCR staining in sham (C) and 1 dpci (D) adult livers. E) Analysis of Atf4 downstream targets upon cryoinjury in Bulk RNA-seq samples of sham and the IA of 1 dpci adult livers. Dashed line: Border zone of the injured area; dpci: days post-cryoinjury; dpf: days-post fertilisation; IA: injured area; vl: ventral lobe. Scale bars: 200 µm.

	Cryoinjury	HPx	Genetic ablation	APAP
**Tissue affected**	Parenchymal and non-parenchymal cells	Apoptosis limited to the tissue around the amputation site	Promoter-specific ablation (Only hepatocytes)	Pericentral hepatocytes and surrounding parenchymal and non-parenchymal cells
**Regeneration**	30	5-7	5	5-7
**Fibrosis**	Yes	No	No	No
**Localised injury response**	Yes	No	No	No
**Regeneration response**	Local hyperplasia & Compensatory growth	Compensatory growth	BECs Transdifferentiation	Compensatory hyperplasia
**Inflammation**	Major	Major	Major	Minor
**Specific transgene**	No	No	Yes	Yes

Table 1. Comparison of liver injury and regeneration methods in animal models.
